# Background phase induced steady‐state effects in velocity quantification using phase‐contrast MRI

**DOI:** 10.1002/mrm.30358

**Published:** 2024-10-24

**Authors:** Carola Fischer, Peter Speier, Tobias Schaeffter, Daniel Giese

**Affiliations:** ^1^ Department of Medical Engineering Technical University of Berlin Berlin Germany; ^2^ Magnetic Resonance Siemens Healthineers AG Erlangen Germany; ^3^ Physikalisch‐Technische Bundesanstalt Braunschweig and Berlin Germany

**Keywords:** background phase, background phase correction, eddy currents, flow quantification, phase‐contrast MRI, steady‐state

## Abstract

**Purpose:**

Flow quantification using phase‐contrast (PC) MRI is based on steady‐state gradient echo (GRE) sequences and is hampered by spatially varying background phase offsets. The purpose of this work was to investigate the effect of steady‐state disruptions during PC‐MRI GRE sequences on these background phases. Based on these findings, a specific sequence and timing is suggested, and caution is expressed when using typical correction algorithms.

**Methods:**

Steady‐state responses in stationary tissue were investigated in different prospectively triggered through‐plane phase‐contrast MRI sequence. Different spoiling methods (gradient spoiling/FISP versus gradient+RF spoiling/FLASH) and interleaving of flow encoding gradients (every TR vs. every ECG cycle) were investigated using simulations, in phantoms and in vivo. Additionally, the effect of relaxation times on the phase offsets was simulated and measured. The impact on image‐ and phantom‐based background phase correction was studied.

**Results:**

Good agreement between simulation and phantom measurements were observed. Different sequences lead to different spatiotemporal and tissue dependent background phases. Average flow rates in the popliteal artery were over‐ and underestimated for ECG‐interleaved and TR‐interleaved FISP acquisitions compared to FLASH, respectively.

**Conclusion:**

Background phase measurements are influenced by steady‐state effects leading to potentially false background phase quantification. Current background phase correction methods cannot correct for the disturbance.

## 1 INTRODUCTION

Phase contrast MRI (PC‐MRI) can measure time‐resolved flow velocities, from which a wide range of hemodynamic parameters can be deduced.^1^, ^2^, ^3^, ^4^, ^5^ It may be applied in the heart, the great vessels, the brain, or abdomen to measure blood flow but was also previously applied to measure hydrodynamics in the CSF.

In PC‐MRI, velocity is encoded into the signals phase by adding bipolar gradients^6^, ^7^ into a steady‐state gradient echo sequence (GRE). These flow encoding/compensating gradients can be alternated every excitation (TR‐interleaved) or every heart cycle (echocardiograph [ECG]‐interleaved). The latter is typically used when temporal resolution is limited by the number of flow encoding steps, for instance in multi‐venc 4D flow^8^ PC‐MRI.

PC‐MRI is known to be hampered by background phase offsets $\phi_{B}$ ^9^:

$$\phi_{B}=\phi_{C}\left(G_{\mathrm{fc}},G_{\mathrm{fe}},\vec{x}\right)+\phi_{E}\left(G_{\mathrm{fc}},G_{\mathrm{fe}},\vec{x},t\right)+\phi_{S}$$

with main contributions from concomitant field phases $\phi_{C}$ and eddy current phases $\phi_{E}$. $\phi_{C}$ can be calculated and subtracted since nominal gradient timings and strengths of the flow compensated $G_{\mathrm{fc}}$ and flow encoded gradients $G_{\mathrm{fe}}$ are known.^10^ In contrast, $\phi_{E}$ induced phase offsets must be corrected by extrapolating phase offsets in static tissue^11^ or repeating the scan with a static phantom.^12^ Typically, this correction is performed on time‐averaged images to increase SNR.

We hypothesize, that there is a third contribution $\phi_{S}$ attributed to steady‐state disruptions throughout the sequence. Alternating the phase offsets originating from $G_{\mathrm{fc}}$ and $G_{\mathrm{fe}}$ violates the GRE steady‐state condition of a constant net phase. Thus, $\phi_{S}$ will be driven by the net background phase induced by $\phi_{C}+\phi_{E}$ and will depend on the encoding pattern as well as the GRE sequence (gradient or gradient+RF‐spoiled, i.e., FISP or FLASH) used. Additionally, tissue relaxation times will likely affect $\phi_{S}$. We further hypothesize, that current background phase correction methods cannot compensate for $\phi_{S}$, which indirectly hampers flow quantification.

To investigate the influence of $\phi_{S}$, we characterized spatio‐temporal and relaxation‐dependent behavior of $\phi_{S}$ with respect to sequence type and encoding timing using simulation, phantom, and in vivo measurements.

## 2 METHODS

### 2.1 PC‐MRI sequence

The research sequence is based on a prospectively ECG‐triggered 2D through‐plane PC‐MRI sequence. FISP contrast was achieved by using spoiling gradients in Read and Slice direction before the next RF pulse, inducing 2π dephasing over a voxel. RF‐spoiled FLASH contrast was achieved by adding a quadratic RF phase increment of 50° every TR.

Flow encoding in slice direction was achieved by using an asymmetric encoding scheme^7^ so that a flow‐encoded and a flow‐compensated acquisition were interleaved. The interleaving was either every TR (*TR‐interleaved*) or every ECG cycle (*ECG‐interleaved*).

A single k‐space line was acquired per heartbeat leading to a doubled scan‐time and halved temporal resolution for the ECG‐interleaved as compared to the TR‐interleaved acquisitions.

**FIGURE 1:**
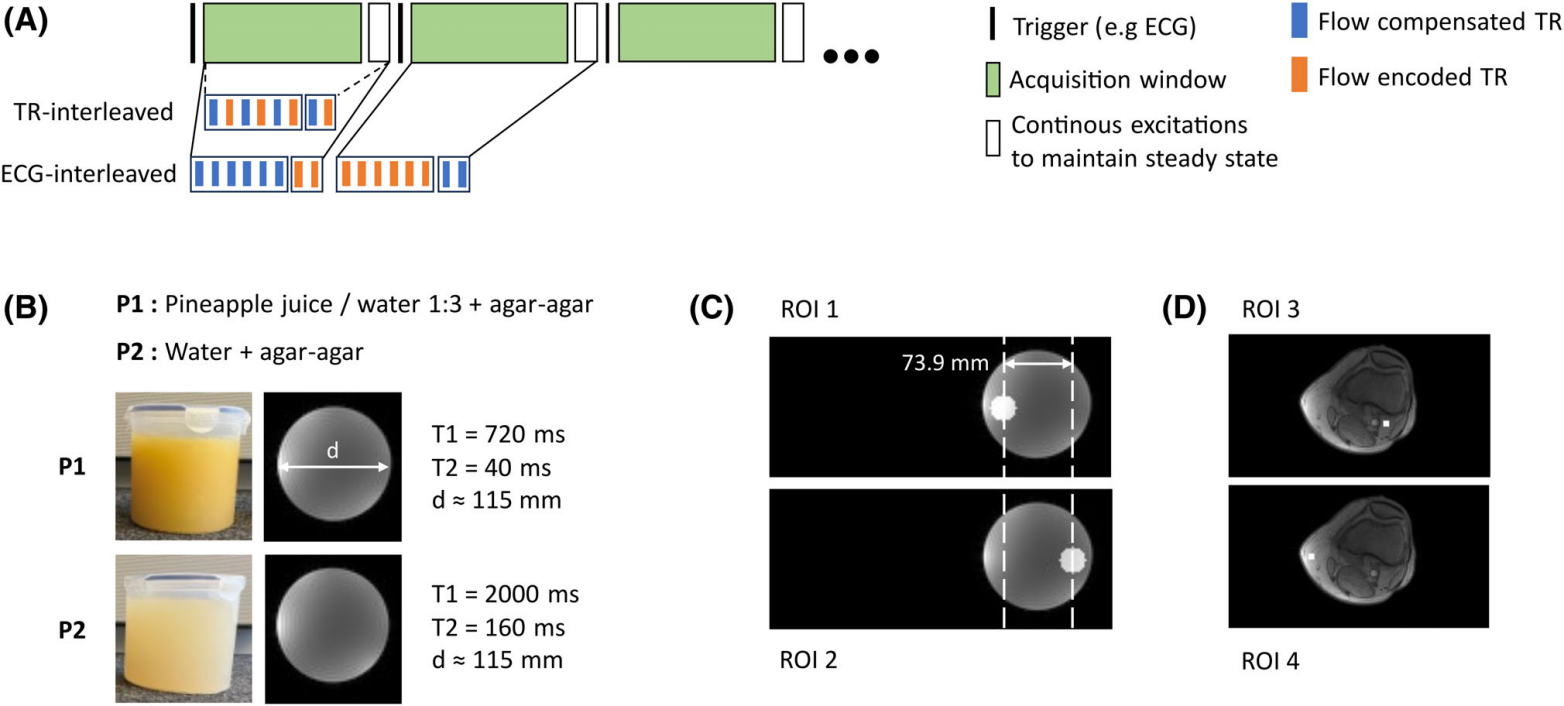
(A) Flow compensated and flow encoded gradient pattern in ECG‐ and TR‐interleaved acquisition schemes. After the acquisition window, RF pulses and gradients continue (steady‐state trigger) to maintain steady‐state. (B) Phantoms P1/P2 and their respective T1/T2 values. (C) Off‐center phantom and ROI positioning. (D) Positioning of regions of interest in in vivo scan.

To maintain the steady‐state of the prospectively triggered sequence, RF excitations and gradients were continuously played out between the end of the acquisition window and the next ECG trigger. This is further referred to as *steady‐state trigger*. In TR‐interleaved acquisitions, the steady‐state trigger continues to alternate between flow compensation and flow encoding until the next ECG trigger. In ECG‐interleaved acquisitions, the steady‐state trigger switches to the next gradient scheme at the end of the acquisition window before the next ECG trigger (see Figure 1A).

The following four sequence modes were investigated: (1) ECG‐interleaved, FISP; (2) ECG‐interleaved, FLASH; (3) TR‐interleaved, FISP; (4) TR‐interleaved, FLASH.

### 2.2 Stationary phantoms

To study spatial, temporal, and relaxation time dependence of $\phi_{S}$, two different gelatinous phantom setups were used (Figure 1B): P1 is a gelatinous homogeneous phantom using agar‐agar and a 1:3 pineapple juice/tap‐water solution (measured T1/T2 = 720 ms/40 ms); P2 is a gelatinous homogeneous phantom using agar‐agar and tap‐water (measured T1/T2 = 2000 ms/160 ms). Both phantoms have a diameter of 115 mm.

### 2.3 Data acquisition and reconstruction

All measurements were acquired on 1.5T systems (MAGNETOM Sola and Altea, Siemens Healthineers AG, Erlangen, Germany). Maximum gradient strengths of the systems are 45 mT/m (Sola) and 33 mT/m (Altea). Slice thickness was 10 mm and the flip angle was 10°. Data were reconstructed offline, using an ESPIRiT^13^ reconstruction implemented in SigPy (v0.1.23).^14^ To investigate all background phase contributions, neither concomitant field correction nor background phase correction was applied.

Stationary phantom scans were acquired using an 18‐channel coil and a venc of 30 cm/s using all four sequence variants. A transversal slice in isocenter was acquired with a FOV of 370 mm in Read‐ and 152.6 mm in Phase‐encode direction. Both phantoms were shifted in‐plane by 120 mm (Figure 1C).

In vivo scans of the popliteal artery in a healthy volunteer were acquired using an 18‐channel knee coil and a venc of 60 cm/s. Informed consent was obtained in accordance with the local authorities and regulations.

Additional acquisition parameters are summarized in the Table S1.

### 2.4 Simulations

Bloch simulations of a single voxel with isochromats were implemented and included varying spoiling schemes, realistic slice profiles, and the effect of flow encoding. The code is implemented in MATLAB R2019b (The MathWorks Inc., Natick, Massachusetts, USA).

Relevant parameters like T1, T2, flip angle, and TR were matched with scanner experiments and phantom properties (Table S1). The slice profile was derived from the Fourier transform of the RF‐pulse.

Background phase offsets were simulated by additional phase rotations of all isochromats in the transverse plane prior to the next RF pulse. This phase offset differed between flow compensating and flow encoding gradients yielding a net phase offset after phase subtraction comparable to background phase measured in PC‐MRI. Background phases originating from the flow compensated acquisition were set to 0 in simulations. Similarly, phase offsets due to flow were neglected. Hence, background phase can be scaled to an arbitrary venc and results are venc‐independent.

TR‐interleaved encoding was achieved by alternating background phase every TR. To simulate ECG‐interleaved encoding, background phase was altered every 150TR, corresponding to an ECG interval of 1020 ms. Steady‐state triggering was applied for 10TR while the last 140TR (952 ms) were defined as the acquisition window (Figure 1A).

For all simulations, background phases from −20%venc to 20%venc were simulated and compared to phantom measurements.

### 2.5 Background phase correction

The impact on flow quantification using image‐based corrections was studied in vivo. Image‐based phase correction was performed as described by Walker et al.^11^ 20% min and 10% max signal thresholds on the magnitude and temporal SD, respectively, were used to mask out static tissue. A second‐order polynomial fit was then performed on the mean phase differences over all heart phases and extrapolated over the entire FOV.

### 2.6 Analysis

First, the spatio‐temporal and tissue‐dependent errors were investigated using simulations and phantom measurements. Regions of interest (ROIs) with a diameter of 27.7 mm (603.5 mm^2^) were analyzed at two different positions, 73.9 mm apart from each other, in the off‐center acquisitions (Figure 1C). Absolute and maximum phase values within the ROIs were computed and the impact on flow quantification was calculated.

In vivo, two ROIs (26 mm^2^) were placed at two different positions within stationary tissue (Figure 1D). The popliteal artery was segmented using Segment v3.2^15^ on one heart phase and used for flow profile analysis. Heart‐phase‐averaged flow rates before and after background phase corrections were calculated.

## 3 RESULTS

### 3.1 ECG‐interleaved PC‐MRI

**FIGURE 2:**
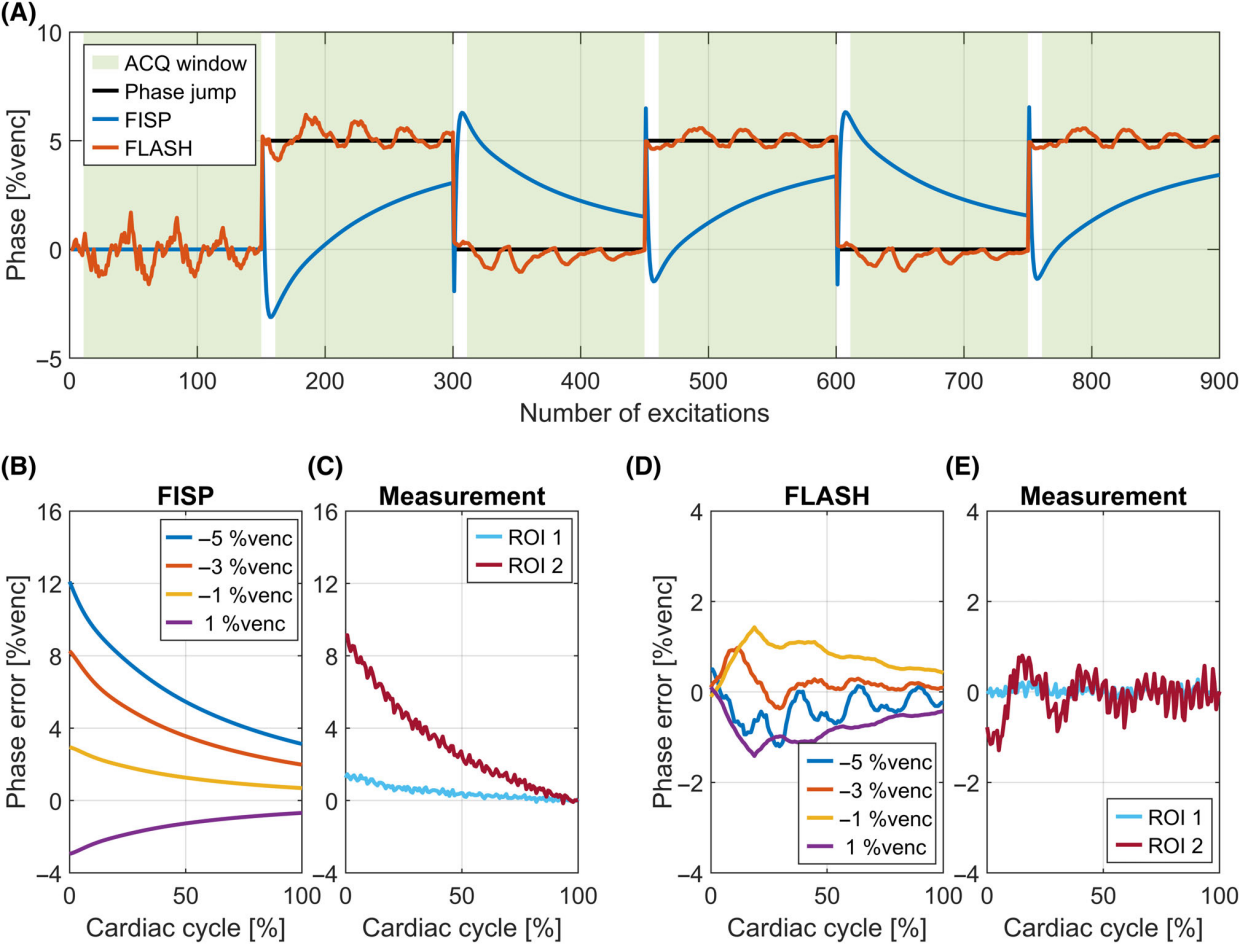
Simulation and measurements of ECG‐interleaved encoding. (A) ECG‐interleaved encoding leads to steady‐state perturbations in both FISP and FLASH contrast. (B–E) After deriving the phase contrast between two consecutive acquisition windows, background phase varies of the cardiac cycle. (B) FISP phase contrast simulations with respect to the input background phase for different input values. (C) Analogous measurements in ROI1 and ROI2 with respect to an average over the last 10 cardiac phases. (D, E) Respective comparison to (B) and (C) using FLASH.

Simulation results in Figure 2A demonstrate steady‐state disruptions when switching between flow encoding and flow compensation every ECG cycle. Although switching occurs at the end of the acquisition window, the disruption causes phase perturbations throughout the ECG cycle. FLASH shows an oscillatory behavior with an overall smaller amplitude as compared to FISP.

Phase contrast calculations derived from the last two simulated ECG cycles are shown for FISP (Figure 2B) and FLASH (Figure 2D) for different background phases. The plots show phase differences to the input background phase. The steady‐state dynamics in FISP lead to a strong temporal background phase response that decays toward the input background phase values without reaching its value. The perturbation is smaller for smaller background phase input values. FLASH produces a smaller oscillatory phase perturbation.

Corresponding FISP and FLASH phantom (P2) measurements are shown in Figure 2C and Figure 2E respectively. As the true background phases are unknown in the measurement, the average value over the last 10 cardiac phases was calculated for FISP and FLASH, respectively, and subtracted from the plots. For FISP, the average values were −0.6%venc (−0.18 cm/s) for ROI 1 and −1.0%venc (−0.30 cm/s) for ROI 2. For FLASH, the averages were −2.8%venc (−0.84 cm/s) for ROI 1 and −6.2%venc (−1.86 cm/s) for ROI 2. The measurements confirm that perturbation is stronger in ROI 2 compared to ROI 1, as background phase offsets are larger. FISP decay and FLASH oscillations are present.

As a result, time‐averaged background phase yields different results for FISP and FLASH using an ECG‐interleaved sequence and may not represent the true background phase. When averaging, FISP yields smaller background phase estimates compared to ground truth background phase offsets. FLASH yields less error.

### 3.2 TR‐interleaved PC‐MRI

**FIGURE 3:**
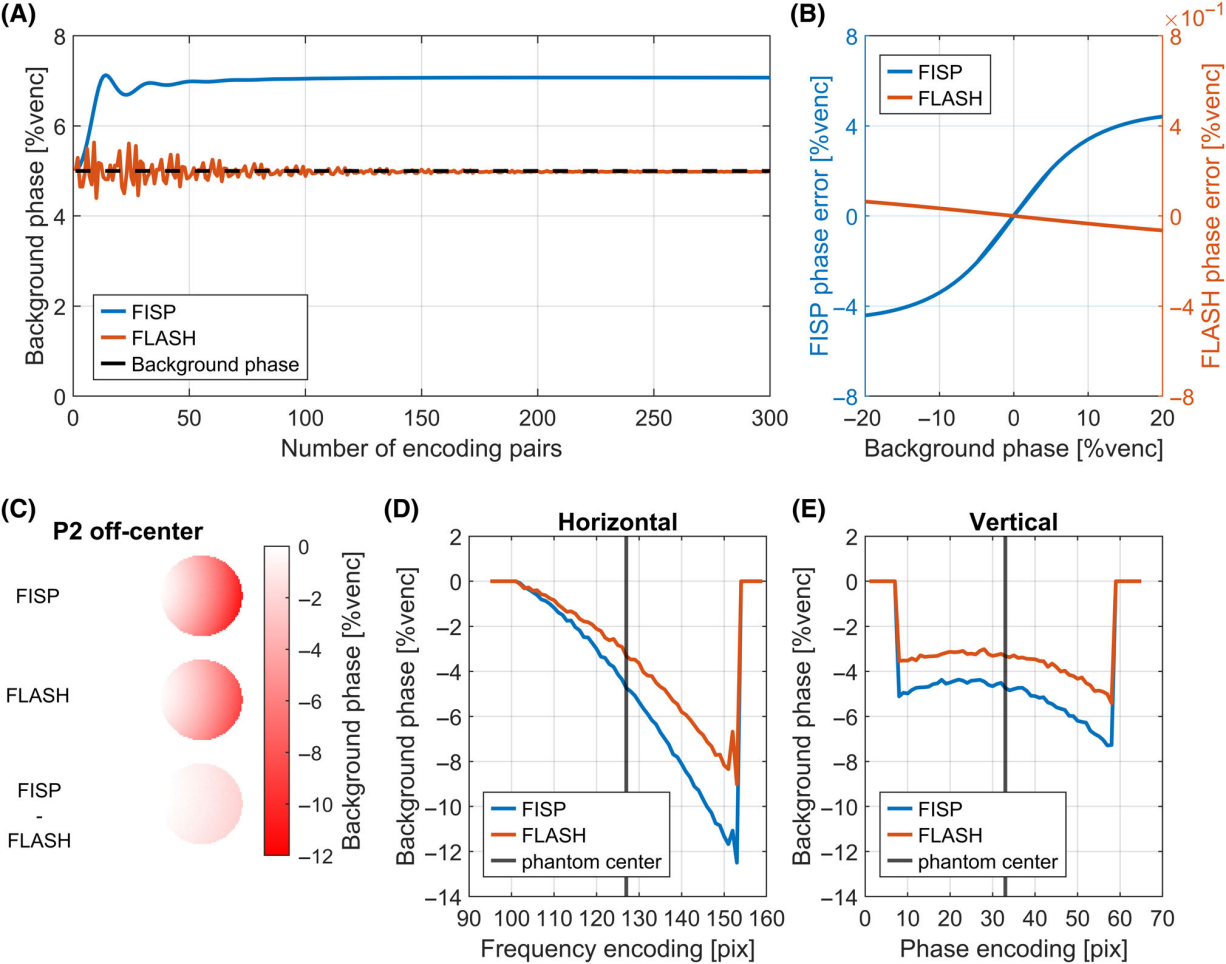
Simulation and measurements of TR‐interleaved encoding. (A) TR‐interleaved encoding in presence of background phase leads to a steady‐state formation after phase‐contrast. (B) Deviations from input background phase scale with background phase strength and are two orders of magnitude stronger for FISP than for FLASH. (C) Phase difference images between FISP and FLASH contrast in P2 off‐center reveal spatial and thereby background strength dependent differences. (D, E) Spatial background phase profiles in P2 for horizontal and vertical profiles through the phantom.

Simulations of TR‐interleaved acquisitions are shown in Figure 3A. In contrast to ECG‐interleaved acquisitions, a phase‐contrast steady‐state is reached after one ECG cycle. For FISP, the reached steady‐state value deviates by 2%venc from background phase input value of 5%venc. This deviation, averaged over the last 20 encoding pairs, increases with input background phase (Figure 3B). Figure 3B also demonstrates that FLASH is less sensitive (<0.06% venc at 20%venc background phase) than FISP. As a result, time‐averaged background phase measurements using a TR‐interleaved FISP sequence will result in over‐ or underestimated background phases depending on the original background phase while the errors when using time‐averaged FLASH background phase is of an order of magnitude smaller.

TR‐interleaved measurements as well as profile plots through P2 are shown in Figure 3C–E. They demonstrate spatial variations as well as differences between FLASH and FISP. Differences increase with increasing background phase, while a similar offset is maintained along the vertical axis due to overall similar background phase offsets. Differences reaching 3.33%venc (1.998 cm/s) are measured along the horizontal profile.

### 3.3 Relaxation time dependence

**FIGURE 4:**
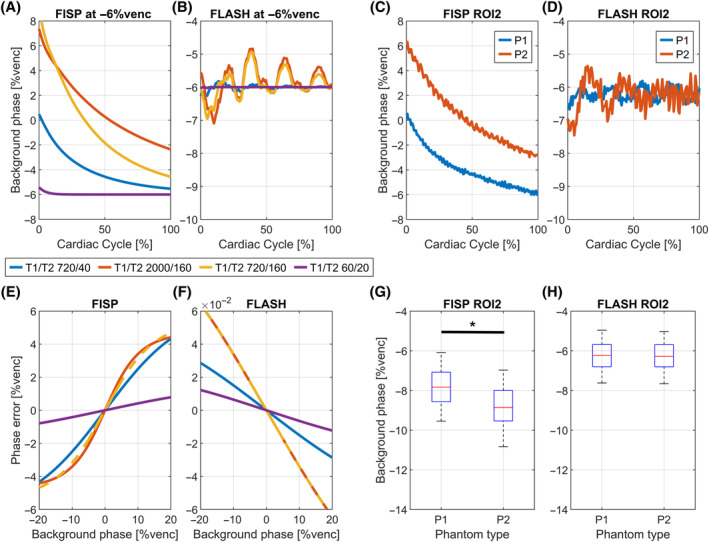
Influence of relaxation time on steady‐state effects in ECG‐interleaved (A–D) and TR‐interleaved (E–H) encoding. (A) Effect of relaxation time on FISP sequence in simulation. (B) Respective comparison for FLASH. (C) FISP behavior in P1 and P2. (D) Respective comparison for FLASH. (E) Error in FISP background phase measurement for different relaxation times compared to simulation input background phase. Simulated background phase at −6%venc is marked for measurement reference. (F) Respective simulation results for FLASH sequence. The error is two orders of magnitude smaller than FISP. (G) Background time‐averaged phase measurements in ROI2 for P1 and P2 using a FISP sequence. Phase measurement differences are statistically significant (*p* < 0.05) between phantoms and comparable to simulated phantom differences at −6%venc. Background phase is overestimated compared to FLASH sequence. (H) Respective comparison for FLASH: Differences are not statistically significant.

Figure 4 analyses the dependence of relaxation times on the previously described effects in ECG‐interleaved (Figure 4A–D) and TR‐interleaved (Figure 4E–H) sequences using simulations and measurements (P1 and P2, ROI2).

Different T1 or T2 alter the perturbation strength and the recovery rate toward the input background phase in ECG‐interleaved sequences (Figure 4A,B). Very short relaxation times reduce the effect. Except for very short relaxation times, none of the simulated or measured values can successfully recover the background phase at the end of the cardiac cycle. Similarly, TR‐interleaved perturbations increase with T1 and T2 (Figure 4E,F). FLASH errors (Figure 4F) remain two orders of magnitude smaller than FISP (Figure 4E).

In ECG‐interleaved FISP measurement, P2 shows stronger perturbations and slower recovery than P1 (Figure 4C). Simulated and measured perturbation strengths for FLASH were similar in P2. In P1, perturbations were stronger in the measurements as compared to the simulations (Figure 4C,D).

Figure 4H confirms measurement differences of background phase between P1 and P2. Measurements differ by 0.99 ± 0.12%venc (0.298 ± 0.036 cm/s). This is statistically significant using a paired t‐test (*p* < 0.05). The phase difference is comparable to a simulated difference of P1 and P2 at 6%venc true background phase. No significant measurement differences were observed for FLASH acquisitions (Figure 4E).

### 3.4 In vivo measurements

**FIGURE 5:**
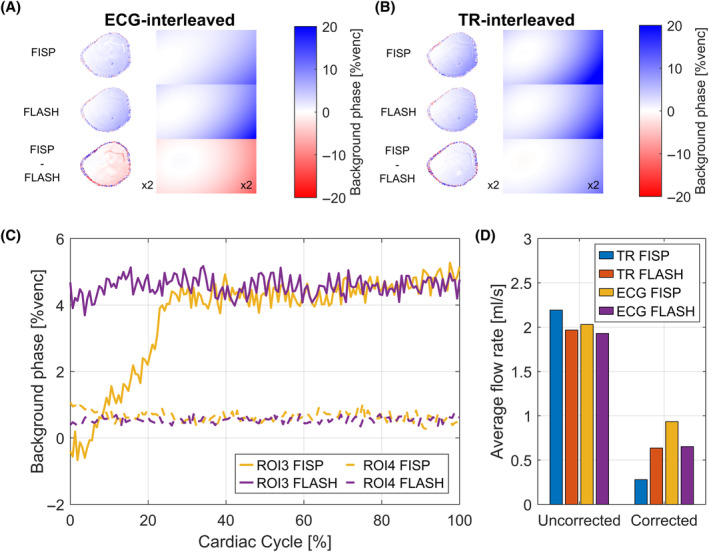
In vivo based background phase correction depends on sequence mode. (A) Comparison of time‐averaged FISP versus time‐averaged FLASH background phase maps and corresponding stationary tissue fits for ECG‐interleaved encoding. The difference image between FISP and FLASH is multiplied by 2 to enhance differences (i.e., 5%venc difference is displayed as 10%venc difference). (B) Analogous comparison for TR‐interleaved encoding. (C) Temporal background phase behavior in stationary tissue for ECG‐interleaved FISP and FLASH, where background phase is strong (ROI 3). (D) Average flow rates in popliteal artery before and after background phase correction.

Figure 5A,B shows second‐order polynomial fits in vivo in all four sequence variants and the respective difference between FISP and FLASH. ECG‐interleaved FISP underestimates background phases (Figure 5A) compared to ECG‐interleaved FLASH and TR‐interleaved FLASH. TR‐interleaved FISP overestimates background phases compared to TR‐interleaved FLASH (Figure 5B). These results are in‐line with simulations and phantom measurements (Figure 2 and Figure 3).

Temporal behavior of the measured background phase in both ROIs for ECG‐interleaved FISP and FLASH is shown throughout the cardiac cycle in Figure 5C.

Figure 5D shows average flow rates in the popliteal artery before and after static fit based background phase correction. Uncorrected flow rates are 2.19 mL/s/1.96 mL/s for TR‐interleaved FISP/FLASH and 2.03 mL/s/1.93 mL/s for ECG‐interleaved FISP/FLASH. After background phase correction is applied, flow rates were 0.28 mL/s/0.63 mL/s and 0.93 mL/s/0.65 mL/s, respectively. Although a larger average flow rate is observed for uncorrected TR‐interleaved FISP, the correction leads to an underestimation of the flow due to background phase overestimation.

## 4 DISCUSSION

Steady‐state dynamics produce background phase contributions. These vary over time, location, with the sequence type used, its timing, and tissue properties.

For an ECG‐interleaved encoding, perturbation occurs every ECG cycle (Figure 2A). The steady‐state may not be reached during each ECG cycle depending on the length of the RR‐interval, the background phase strength $\phi_{C}+\phi_{E}$ (Figure 2B–E) and the tissue relaxation properties (Figure 4A–D). ECG‐interleaved FISP is more affected than ECG‐interleaved FLASH. This was confirmed in simulation and phantom measurements (Figures 2/4A–D).

In contrast, TR‐interleaved acquisitions can be assumed to reach a steady‐state, especially when dummy‐ECG cycles are used. Although, background phase oscillates within every RF‐cycle, the phase difference reaches a steady‐state. The steady‐state value differs for TR‐interleaved FISP and TR‐interleaved FLASH and scales with the original background phase strength $\phi_{C}+\phi_{E}$ (Figure 3A,B). Simulations show that absolute $\left|\phi_{S}\right|$ is generally stronger for FISP than for FLASH. TR‐interleaved FLASH can therefore be considered robust against steady‐state perturbations (≪0.4%venc^9^).

It was demonstrated that ECG‐interleaved and TR‐interleaved background phases vary with tissue relaxation times (Figure 4). Longer T1 or T2 amplify the perturbation, which led to measurable differences between P1 and P2, for all sequences except TR‐interleaved FLASH acquisitions.

FLASH results in a smaller effect than FISP due to the effect of RF‐spoiling damping the steady‐state transient state.

The described steady‐state induced background phase offsets $\phi_{S}$ cannot be corrected using the typical postprocessing strategies:

Using a phantom‐based correction,^12^ the choice of the relaxation times is critical. Very short relaxation times are useful (Figure 4) to suppress $\phi_{S}$. However, $\phi_{S}$ will still be present in the actual scan. As the goal of the phantom‐based correction is to reproduce $\phi_{S}$ with the phantom, matching the relaxation times of the tissue of interest (e.g., blood, CSF, …) might be alluring. Nevertheless, the effect of (pulsatile) flow is known to alter $\phi_{S}$.

Using a tissue fit correction^11^ suffers from similar shortcomings. As $\phi_{S}$ depends on relaxation times, it cannot be assumed that the pixels used for the fit have identical relaxation properties as the pixel in which flow is quantified. The extrapolation is therefore prone to errors. Additionally, $\phi_{S}$ will be altered by flow‐dependent effects (in‐flow, phase disturbances) leading to wrong extrapolation assumptions. As shown in vivo, such time‐averaged tissue fit corrections lead to different results for different sequences (Figure 5D) and different relaxation times. Especially, in presence of strong inflow, steady‐state effects in the vessels are minimized. In the present measurements, TR‐interleaved FISP led to lower flow rates compared to FLASH sequences after correction. ECG‐interleaved FISP led to larger flow rates because temporal steady‐state perturbations (Figure 5C) were time‐averaged for the tissue fit (Figure 5A). In conclusion, a time‐resolved tissue fit might worsen flow quantification results, because $\phi_{S}\left(t\right)$ is different in stationary tissue as compared to the vessel of interest.

To solve the described issue, $\phi_{S}$ must be known or minimized. Due to the dependencies of $\phi_{S}$ on $\phi_{C}+\phi_{E}$, sequence timing, relaxation times, flow profiles, the knowledge of the exact $\phi_{S}$ within the vessels of interest is extremely challenging and, in our opinion, impossible.

To minimize $\phi_{S}$, both main contributions $\phi_{E}$ and $\phi_{C}$ should be minimized. In our measurements, the main contribution to $\phi_{S}$ are concomitant field phases, mainly due to the asymmetric flow encoding used. The effect is therefore expected to increase with lower field strengths and stronger gradients.^10^ Using a symmetric flow encoding scheme would remove concomitant field contributions^10^ but come with other drawbacks (longer TEs, potentially stronger eddy‐currents). To minimize $\phi_{E}$, an optimal eddy‐current pre‐emphasis should be used and all effects potentially leading to stronger eddy‐current background phases (acoustic resonances, mechanical vibrations, temperature changes) minimized. Alternatively, optimizing TE^16^ or spoiler gradients^17^ might help to minimize $\phi_{E}$.

In general, central positioning of the vessel is beneficial as $\phi_{E}$ and $\phi_{C}$ increase with the distance from the isocenter.^10^, ^18^

FLASH‐based PC‐MRI have shown the smallest errors from $\phi_{S}$ in simulations, phantom and in vivo measurements. TR‐interleaved FLASH appears robust with negligible errors.

### 4.1 Limitations

A limitation of our study is that we did not investigate the effect of flow on steady‐state behavior. Phase contributions from flow may introduce additional phase errors as flow phase also alternates with encoding gradients. We expect, this effect is stronger in presence of lower flow velocities or large volumes (e.g., 4D flow), when magnetization stays within the excited volume for several excitations. Pulsatile flow might additionally introduce phase contributions due to changes in phase.

As seen in Figures 2C/E and 4C,D, phantom measurements were likely affected by table vibrations. Phase oscillations in the order of 15–30 Hz in the ECG‐interleaved sequences were observed. These vibrations might additionally affect steady‐state dynamics and can be stronger than the measured steady‐state disturbances.

We only investigated through‐plane encoded PC‐MRI. Phase behavior might change for in‐plane encoding. Especially in the phase‐encode direction, different dynamics are expected, as the zeroth moment is typically rephased and not spoiled.^6^

Finally, the measurements are non‐accelerated and prospectively triggered. The effect of retrospective gating has not been studied. However, we suspect no significant changes in TR‐interleaved acquisitions. ECG‐interleaved perturbances should be worse since no additional steady‐state triggers are applied; however, cardiac phase interpolation might alter the effect. Similarly, acceleration techniques such as segmented k‐space or parallel imaging were not investigated. Although acceleration might average the effect over several k‐space lines (ECG‐interleaved), it should be identical for TR‐interleaved acquisitions using identical reordering patters. Generally, the effect of reordering on background phase behavior should be further investigated.

## 5 CONCLUSIONS

Background phase measurements depend on the steady‐state GRE sequence and on the interleaving type of the flow encoding (TR‐ or ECG‐interleaved). The error is due to a violation of steady‐state conditions due to alternating background phase offsets (concomitant fields and eddy currents). While TR‐interleaved FLASH acquisitions are less affected, both ECG‐interleaved FISP and FLASH lead to false and temporally varying background phases. TR‐interleaved FISP is temporally stable but also results in false background phases. As all effects are due to steady‐state violations, the tissues relaxation times have an important effect on their strengths which has implications on the typically used correction strategies. Flow quantification may therefore be hampered.

We suggest that steady‐state dynamics should be considered if new PC‐MRI sequences are developed (e.g., PC‐bSSFP). The investigation of in‐plane flow encoding as well as multi‐venc or multi‐dimensional flow encoding on the steady‐state behavior is warranted. Finally, the effect of flow effects on the steady‐state is of major importance and will be investigated in future work.

## CONFLICT OF INTEREST STATEMENT

Carola Fischer received a salary by Siemens Healthineers AG during the course of her PhD. Peter Speier and Daniel Giese are employees of Siemens Healthineers AG.

## Supporting information


**Table S1.** Acquisition parameters of in vivo and phantom study. Respective simulation parameters (compared to phantom study).
