# Supplementary material for: Background phase induced steady‐state effects in velocity quantification using phase‐contrast MRI
Source: Magn Reson Med. 2024 Oct 24;93(4):1690–9. doi: 10.1002/mrm.30358 (PMC11782709; doi:10.1002/mrm.30358)
Supplement: Supplementary file 1 — Table S1. Acquisition parameters of in vivo and phantom study. Respective simulation parameters (compared to phantom study). [file MRM-93-1690-s001.docx]

# Supplemental Material for Technical Note:

## Background phase induced steady-state effects in velocity quantification using phase-contrast MRI

**Supplemental Table S1:** Acquisition parameters of in vivo and phantom study. Respective simulation parameters (compared to phantom study).

| Parameter | Simulation | Phantom | Healthy Volunteer |
| --- | --- | --- | --- |
| In-plane Resolution [mm^2] | x | 2.31 x 2.31 | 1.70x1.70 |
| TR [ms] | 6.8 | 6.77 | 5.54 |
| TE [ms] | x | 4.60 | 3.45 |
| Venc [cm/s] | 30 | 30 | 60 |
| Acquisition Window [ms] | 952 (140TR) | 950 | 900 |
| Mean RR-Intervall [ms] | 1020 (150TR) | 1000 | 1038 / 986  (ECG- /TR-interleaved) |
